# Autopromotion of K-Ras4B Feedback Activation Through an SOS-Mediated Long-Range Allosteric Effect

**DOI:** 10.3389/fmolb.2022.860962

**Published:** 2022-04-08

**Authors:** Xuan He, Kui Du, Yuanhao Wang, Jigang Fan, Mingyu Li, Duan Ni, Shaoyong Lu, Xiaolan Bian, Yaqin Liu

**Affiliations:** ^1^ Department of Pharmacy, Ruijin Hospital, Shanghai Jiao Tong University, School of Medicine, Shanghai, China; ^2^ School of Chemistry and Chemical Engineering, Shaoxing University, Shaoxing, China; ^3^ Department of Pathophysiology, Key Laboratory of Cell Differentiation and Apoptosis of Chinese Ministry of Education, School of Medicine, Shanghai Jiao Tong University, Shanghai, China; ^4^ The Charles Perkins Centre, University of Sydney, Sydney, NSW, Australia; ^5^ Medicinal Chemistry and Bioinformatics Center, Shanghai Jiao Tong University, School of Medicine, Shanghai, China

**Keywords:** K-Ras4B, SOS, allosteric regulation, molecular dynamics simulation, small-molecule interactions

## Abstract

The Ras-specific guanine nucleotide exchange factors Son of Sevenless (SOS) regulates Ras activation by converting inactive GDP-bound to active GTP-bound states. The catalytic activity of Ras is further allosterically regulated by GTP−Ras bound to a distal site through a positive feedback loop. To address the mechanism underlying the long-range allosteric activation of the catalytic K-Ras4B by an additional allosteric GTP–Ras through SOS, we employed molecular dynamics simulation of the K-Ras4B^G13D^•SOS^cat^ complex with and without an allosteric GTP-bound K-Ras4B^G13D^. We found that the binding of an allosteric GTP−K-Ras4B^G13D^ enhanced the affinity between the catalytic K-Ras4B^G13D^ and SOS^cat^, forming a more stable conformational state. The peeling away of the switch I from the nucleotide binding site facilitated the dissociation of GDP, thereby contributing to the increased nucleotide exchange rate. The community networks further showed stronger edge connection upon allosteric GTP−K-Ras4B^G13D^ binding, which represented an increased interaction between catalytic K-Ras4B^G13D^ and SOS^cat^. Moreover, GTP−K-Ras4B^G13D^ binding transmitted allosteric signaling pathways though the Cdc25 domain of SOS that enhanced the allosteric regulatory from the K-Ras4B^G13D^ allosteric site to the catalytic site. This study may provide an in-depth mechanism for abnormal activation and allosteric regulation of K-Ras4B^G13D^.

## 1 Introduction

As the central control element of the signal transduction pathway, GTPases and their related regulatory factors and effectors are involved in almost all aspects of cell biology [(Colicelli, 2004; Mitin et al., 2005; Bos et al., 2007)]. Ras superfamily proteins, the classical members of small GTPases, possess a shared biochemical activity: GTP (guanosine triphosphate) binding and hydrolysis. Three major isoforms, including H-Ras, K-Ras, and N-Ras, are closely related due to high overall sequence identity [(Prior et al., 2012), (Cox et al., 2014)]. Among them, K-Ras covers 85% mutated isoforms in Ras-driven cancers [(Fernandez-Medarde and Santos, 2011; Stephen et al., 2014)]. Ras transforms between inactive GDP-bound and active GTP-bound states, where only the Ras−GTP complex can activate downstream signaling pathways *via* high-affinity binding to its effectors (Bourne et al., 1990; Qiu et al., 2021a). The oncogenic forms of Ras are manifested as the impaired GTPase cycle because of the exchange dysregulation of Ras–GTP hydrolysis/guanine nucleotide (Lu et al., 2015; Parker and Mattos, 2018). The continuous activation of Ras contributes to several malignant phenotypes such as cell differentiation and proliferation (Drosten et al., 2010). The nucleotide binding pocket of Ras consists of three functional regions including the P-loop [residues (Milburn et al., 1990; Vetter and Wittinghofer, 2001; Drosten et al., 2010; Goitre et al., 2014; Lu et al., 2015; Simanshu et al., 2017; Parker and Mattos, 2018; Lu et al., 2019a)], switch I [residues (Sondermann et al., 2004; Freedman et al., 2006; Ni et al., 2017; Ni et al., 2018; Lu et al., 2019b; Ni et al., 2020; Fan et al., 2021; Li et al., 2021; Moghadamchargari et al., 2021)], and switch II [residues (Boykevisch et al., 2006; Newman, 2006; Krissinel and Henrick, 2007; Eargle and Luthey-Schulten, 2012; Vo et al., 2016; Li et al., 2018; Bandaru et al., 2019; Wang et al., 2019; Li et al., 2020; Aledavood et al., 2021; Feng et al., 2021; Foutch et al., 2021; He et al., 2021; Liang et al., 2021; Liu et al., 2021; Marasco et al., 2021; Ni et al., 2021; Okeke et al., 2021)] (Milburn et al., 1990; Vetter and Wittinghofer, 2001; Goitre et al., 2014). The structures of GDP-bound and GTP-bound forms mainly differ by the conformation of switches I and II, both of which are highly dynamic regions. The activation of Ras requires guanine nucleotide exchange factors (GEFs), facilitating the exchange of GDP to GTP, while its inactivation is terminated by GTPase-activating proteins (GAPs) stimulating the hydrolysis of intrinsic GTP (Simanshu et al., 2017; Lu et al., 2019a; Jang et al., 2020).

The multiprotein, Son of Sevenless (SOS), is a GEF consisting of ∼1,330 residues (Gureasko et al., 2008; Rojas et al., 2011). The core catalytic region of SOS, which is required for Ras-specific nucleotide exchange activity, consists of a Ras exchanger motif (Rem) domain and a Cdc25 domain, termed SOS^cat^ (residues 551–1050) (Chardin et al., 1993). Grb2 recruits SOS to the plasma membrane to initiate SOS activation [(Kazemein Jasemi et al., 2021)]. In general, the interaction of SOS with Ras–GDP promotes the disassociation of Ras and GDP molecules, and the nucleotide-free Ras is accessible to bind more cellular GTP (Lu et al., 2016a). It has been proposed that the catalyzed conversion of Ras by SOS covers a multi-step mechanism, which can be roughly divided into the following processes: 1) the exchange reaction is initiated by the formation of a low-affinity complex containing an inactive Ras−GDP and activated SOS; 2) the Ras active site opens widely and expels GDP, thereby forming a high-affinity binary Ras•SOS complex. Ras has a picomolar high affinity to both GDP and GTP, and the tightly bound nucleotides must turn loose for their fast dissociation (Goody et al., 1991). According to experimental evidence, the reverse isomerization reaction from high- to low-affinity bound conformation of the Ras–GDP complex may be one rate-limiting step during the SOS-catalyzed exchange (Lenzen et al., 1998). The structure of the Ras•SOS^cat^ complex showed that the helical hairpin region protruding from the main body of the Cdc25 domain of the SOS^cat^ is inserted between the switch I and II regions of Ras protein, opening the Ras nucleotide binding site for GDP release (Hall et al., 2001). In addition, Ras is unstable in the absence of nucleotide, and SOS stabilizes its nucleotide-free form by forming a large interface with the switch II, which provides the main anchor for the interactions of SOS with Ras. The formation of this extensive interface is probably crucial for stabilizing the unstable nucleotide-free Ras and protect it from unfolding (Cherfils and Zeghouf, 2013); 3) since the cellular concentration of GTP is high, GTP binds to the nucleotide-free Ras (Buday and Downward, 2008); and 4) after completing the exchange from GDP to GTP, the binary GTP-bound Ras complex releases from the SOS, yielding the active GTP-bound Ras form. Removal of the bound nucleotide resulted in a stable complex, which was key for obtaining many crystal structures of nucleotide-free Ras•SOS complexes and had been deeply investigated during the last decades (Cherfils and Zeghouf, 2013).

Multiple crystal structures of Ras under different nucleotide-bound states with SOS have been determined, providing comprehensive details on the activation mechanism of Ras (Chardin et al., 1993; Bos et al., 2007). Interestingly, Margarit et al. (2003) reported an H-Ras^A59G^•SOS^cat^ structural complex and found an additional Ras−GTP located at the Rem and Cdc25 domains of SOS^cat^. The resulting mutant structure is an Ras•SOS^cat^•Ras−GTP ternary complex, consisting of two H-Ras^A59G^ molecules (one is nucleotide-free catalytic H-Ras^A59G^ and the other is GTP-bound allosteric H-Ras^A59G^) and one SOS^cat^ molecule (Margarit et al., 2003). Given that SOS is allosterically activated by Ras−GTP, multiple studies have revealed a conformational switch of SOS induced by Ras−GTP and the change of catalytic activity in the ternary system (Freedman et al., 2006). A positive feedback mechanism has been reported in the activation of Ras by SOS (Sondermann et al., 2004). However, the interaction between the oncogenic Ras mutants and SOS remains insufficiently characterized, and the precise structural details of the positive feedback mechanism are still unknown.

Recently, a structure of the K-Ras4B^G13D^•SOS^cat^•K-Ras4B^G13D^−GTP complex was resolved. Biochemical data implied that K-Ras4BG13D−GTP binding allosterically increased the nucleotide exchange rate of K-Ras4B at the active site and led to the reposition of the switch I and II regions of K-Ras4B^G13D^ (Moghadamchargari et al., 2021). This brought a partial understanding of the interaction between oncogenic Ras mutants and SOS. However, the mechanism underlying the long-range allosteric activation of the catalytic K-Ras4B by an additional allosteric GTP–Ras through SOS remains poorly understood. Thus, further exploration of the allosteric regulation of Ras–GTP can uncover more information pertaining to the activation mechanism of Ras and may provide an avenue for drug discovery (Lu et al., 2019b; Fan et al., 2021).

Conformational dynamics of signaling proteins are vital for the realization of their biological functions (Ni et al., 2017; Ni et al., 2018; Ni et al., 2020; Lu et al., 2021a; Qiu et al., 2021b; Li et al., 2021). In this study, using explicit molecular dynamics (MD) simulations, we investigated the mechanism of allosteric activation of the catalytic Ras located at the SOS^cat^ active site induced by the allosteric Ras–GTP binding at the SOS^cat^ distal site. We established the K-Ras4B^G13D^•SOS^cat^ binary system and the K-Ras4B^G13D^•SOS^cat^•K-Ras4B^G13D^–GTP ternary system to explore conformational changes upon allosteric K-Ras4B^G13D^–GTP binding. The results mainly focused on dynamic conformational changes of the catalytic K-Ras4B^G13D^ and allosteric pathways. In both structural and energetic aspects, binding of K-Ras4B^G13D^–GTP enhanced the affinity between the catalytic K-Ras4B^G13D^ and SOS^cat^, yielding a more stable structural state. Furthermore, the variation of the K-Ras4B^G13D^ switch I region caused the expansion of the nucleotide binding pocket, which contributed to the increased nucleotide exchange rate upon K-Ras4B^G13D^–GTP binding. This study may provide insights into the allosteric regulation of the K-Ras4B^G13D^•SOS^cat^ complex by K-Ras4B^G13D^–GTP.

## 2 Materials and Methods

### 2.1 Construction of Simulated Systems

Two simulated systems were established, including the K-Ras4B^G13D^•SOS^cat^ binary system and the K-Ras4B^G13D^•SOS^cat^•K-Ras4B^G13D^–GTP ternary system, based on the K-Ras4B^G13D^•SOS^cat^•K-Ras4B^G13D^–GppNHp crystal structure (PDB ID: 7KFZ) (Moghadamchargari et al., 2021). The missing residues were remodeled using Discovery Studio and GppNHp was replaced by a GTP molecule. By deleting K-Ras4B^G13D^–GTP in the ternary system, the K-Ras4B^G13D^•SOS^cat^ binary system was extracted from the K-Ras4B^G13D^•SOS^cat^•K-Ras4B^G13D^–GTP complex.

### 2.2 MD Simulations

The initial parameter files for minimization and simulation were prepared using Amber18 package with the ff14SB force field (Maier et al., 2015) and the general Amber force field (GAFF) (Wang et al., 2004; Lu et al., 2016b; Wang et al., 2021a). Both complexes were solvated in a truncated octahedron transferable intermolecular potential three-point (TIP3P) water box, and then Na^+^ and Cl^−^counterions were added to neutralize the system and to mimic a simulated body fluid (Jorgensen et al., 1983), (Wang et al., 2022). Next, both systems were processed by two rounds of energy minimizations using steepest descent and conjugate gradient minimization steps. After that, both systems’ temperatures increased from 0 to 300 K in 300 ps in a canonical ensemble (NVT), followed by the equilibration runs of 700 ps in the NVT ensemble. Finally, three independent rounds of 1 μs MD simulations were performed for both systems in the isothermal and isobaric ensembles (NPT) with the periodic boundaries condition. In the course of MD simulations, long-range electrostatic interactions were calculated using the particle mesh Ewald (PME) method [(York et al., 1994), (Pal et al., 2021)], while short-range electrostatic interactions and van der Waals interactions were defined using a cutoff distance of 10 Å. Covalent bonds involving hydrogen were constrained using the SHAKE method [(Ryckaert et al., 1977; Byun et al., 2020; Liang et al., 2020)].

### 2.3 Principal Component Analysis

PCA was applied to capture the essential motions and characterize the overall dominant conformational transitions in two systems [(Hernández-Alvarez et al., 2021)]. In order to describe the motions of the system, the covariance matrix of Cα atoms was diagonalized to create a new set of eigenvectors (also called PC) in PCA. The eigenvalue of each PC was related to the mean square fluctuation of the PC projected by the trajectory of the entire system. Hence, the first ranked PC (PC1) corresponded to the most dominant amplitude movement within the system, and the system dynamics projected along PC1 was defined as “essential dynamics” [(Amadei et al., 1993)]. In this work, PCA results were conducted on the 2D plane according to the PC1 and PC2 to determine the major conformational dynamics of K-Ras4B^G13D^•SOS^cat^. After that, cluster analyses were used to extract the most representative conformations from PC1 to PC2, which were superimposed using all Cα atoms prior to eliminate the overall rotation and transition [(Shao et al., 2007)].

### 2.4 Molecular Mechanics Poisson–Boltzmann Surface Area Calculations

The binding free energies between K-Ras4B^G13D^ and SOS^cat^ in both systems were calculated using the MM/PBSA plugin from MMPBSA.py in Amber18 package (Chong et al., 2009; Shibata et al., 2020; Wang et al., 2020; An et al., 2021). The binding free energy $\Delta G_{binding}$ was defined based on the following equation:

$$\Delta G_{binding}=G_{complex}-G_{ligand}-G_{receptor}.$$ (1)

According to the second thermodynamic law, $\Delta G_{binding}$ equals to the enthalpy changes ( ΔH ) minus the product of the entropy changes and temperature ( TΔS ). Furthermore, ΔH can be divided in to the molecular mechanical energy ( $\Delta E_{MM}$ ) and solvation energy change ( $\Delta G_{solv}$ ). Therefore, $\Delta G_{binding}$ could be calculated as follows:

$$\Delta G_{binding}=\Delta E_{MM}+\Delta G_{solv}-T\Delta S.$$ (2)

$\Delta E_{MM}$ mainly consists of three parts: a van der Waals component $\Delta E_{vdW}$ , an electrostatic component $\Delta E_{ele}$ , and an intramolecular energy component $\Delta E_{\mathrm{int}}$ .

$$\Delta E_{MM}=\Delta E_{vdW}+\Delta E_{ele}+\Delta E_{\mathrm{int}}.$$ (3)

For the $\Delta G_{solv}$ term in Eq. 2, the Poisson–Boltzmann continuum solvent model was used for calculation, which was further divided into the polar part $\Delta E_{PB}$ and the non-polar part $\Delta E_{nonpolar}$ :

$$\Delta G_{solv}=\Delta E_{PB}+\Delta E_{nonpolar}.$$ (4)

The non-polar component $\Delta E_{nonpolar}$ can be calculated using the solvent-accessible surface area (SASA) from Eq. 5. The constant γ and solvation parameter b were 0.00542 kcal·mol^−1^·Å^−2^ and 0.92 kcal/mol, respectively.

$$\Delta E_{nonpolar}=\gamma SASA+b.$$ (5)

The conformation entropy component ( −TΔS ) can be calculated using normal mode analysis with a quasi-harmonic model, but it could be omitted here because of the similarity within the K-Ras4B^G13D^•SOS^cat^ complex between two systems. In addition, considering that the calculation of entropy term required a relatively long period, we omitted the −TΔS term in our calculations (Liang et al., 2021). To improve the predictive accuracy, the internal dielectric constant was set to 4.0 (Li et al., 2018).

### 2.5 Dynamic Network Analysis

In order to characterize the inter-residue correlations, all protein Cα atoms were calculated using the correlation coefficient *C*_*ij*_, which reflected the correlated degree of motions of Cα atoms (Wang et al., 2019; Aledavood et al., 2021; He et al., 2021; Marasco et al., 2021; Okeke et al., 2021). *C*_*ij*_ was calculated as follows:

$$C_{i,j}=\frac{\langle \left(r_{i}-r_{i}\right)\rangle \cdot \langle \left(r_{j}-r_{j}\right)\rangle }{\sqrt{\langle \left(r_{i}-r_{i}^{2}\right)\rangle \cdot \langle \left(r_{j}-r_{j}^{2}\right)\rangle }},$$ (6)

where *r*_*i*_ and *r*_*j*_ represent the positions of the *i*th and *j*th Cα atoms.

To calculate the community network *via* the NetworkView plugin in VMD (Eargle and Luthey-Schulten, 2012), the Cα atom of each residue was recognized as a node, and edges were formed when two nodes stayed within a cutoff distance of 4.5 Å for at least 75% of the simulation time. To weight edges and describe the edge distance, the correlation data can be further calculated using the following equation:

$$d_{i,j}=-\log\left(\left|C_{i,j}\right|\right).$$ (7)

The community network was defined and optimized using the Girvan–Newman algorithm based on the information of betweenness (Newman, 2006), (Liu et al., 2021). In addition, the shortest paths were calculated using the Floyd–Warshall algorithm as cross-community communication (He et al., 2021). Suboptimal pathways were defined as within 20 Å to the optimal pathway (69).

## 3 Results

### 3.1. RMSD and RMSF Analysis

**FIGURE 1:**
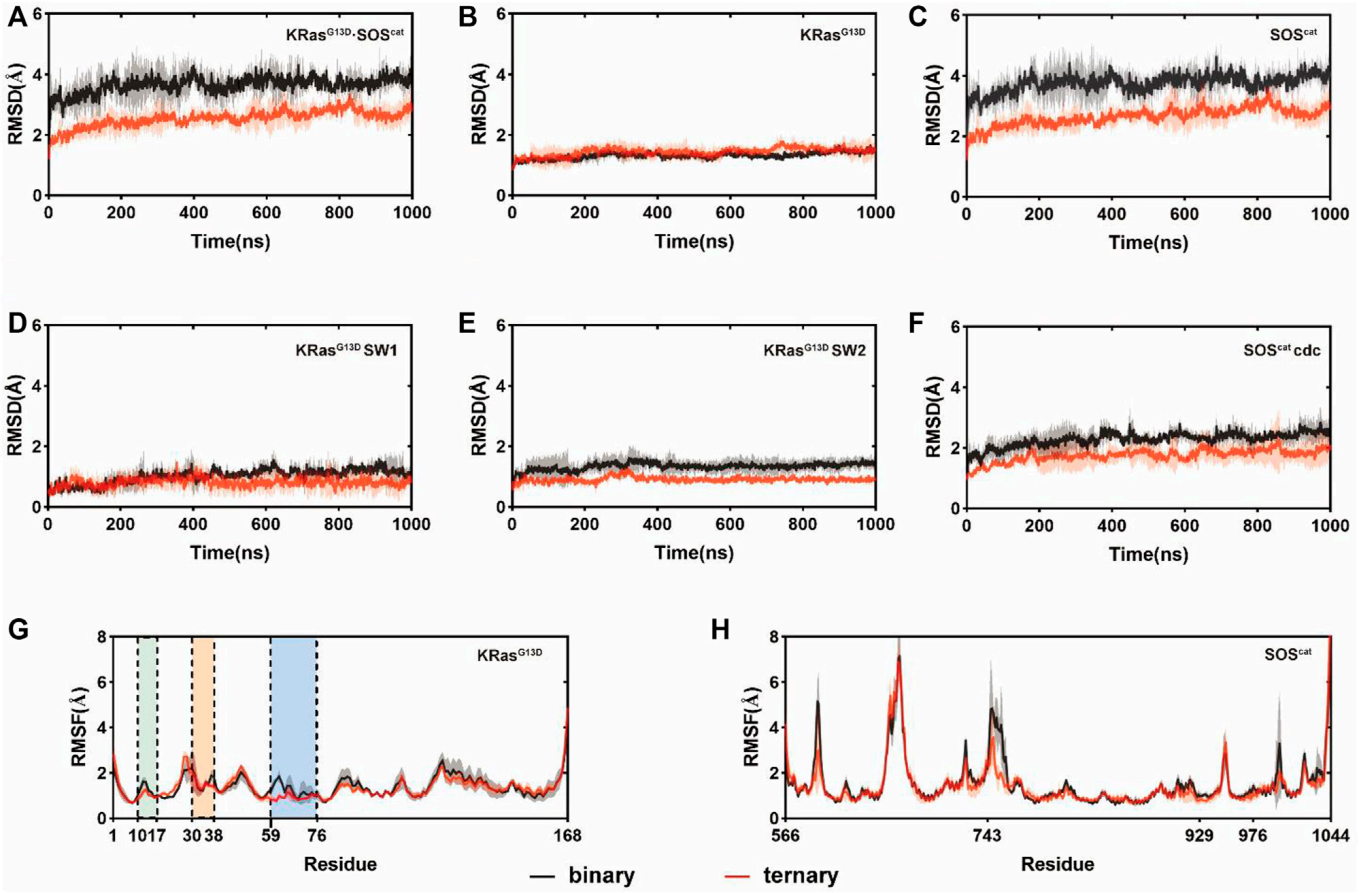
Conformational dynamics of the K-Ras4B^G13D^•SOS^cat^ complex in the binary and ternary systems. RMSDs of Cα atoms in the binary (black) and ternary (red) systems within **(A)** all K-Ras4B^G13D^•SOS^cat^ complex residues; **(B)** K-Ras4B^G13D^ residues; **(C)** SOS^cat^ residues; **(D)** K-Ras4B^G13D^ switch I residues; **(E)** K-Ras4B^G13D^ switch II residues, and **(F)** SOS^cat^ Cdc25 domain residues. RMSFs of Cα atoms in the binary (black) and ternary (red) systems within **(G)** K-Ras4B^G13D^ residues and **(H)** SOS^cat^ residues. The P-loop, the switch I and switch II regions are marked by green, orange, and blue backgrounds, respectively. Gray and red transparencies represent the standard deviations.

To capture the dynamic conformational changes, we conducted three independent rounds of 1 μs MD simulations on both binary and ternary complexes. Root-mean-square deviations (RMSDs) of Cα atoms in each residue of K-Ras4B^G13D^ **•**SOS^cat^ were analyzed in the two systems relative to the initial structures, which reflected the conformational dynamics over simulations. K-Ras4B^G13D^ **•**SOS^cat^ in both systems reached equilibrium after ∼200ns MD simulations. The RMSD values of K-Ras4B^G13D^ **•**SOS^cat^ in the binary and ternary systems were 3.65 ± 0.26 Å and 2.55 ± 0.27 Å, respectively, which indicated some conformational discrepancies between two systems (Figure 1A). Meanwhile, we separately calculated the RMSDs of K-Ras4B^G13D^ and SOS^cat^. As shown in Figures 1B,C, the RMSD of SOS^cat^ in the ternary system was notably lower than that in the binary system, while the RMSD of K-Ras4B^G13D^ showed slight conformational differences between two systems. Then, we further explored the specific functional regions of K-Ras4B^G13D^ and SOS^cat^. Focusing on the switch I and switch II regions, we found that the discrepancies of K-Ras4B^G13D^ mainly contributed by the switch II region (Figures 1D,E). However, the RMSD of the Cdc25 domain of SOS^cat^ showed significant discrepancies between two systems, which led to the overall conformational differences in the K-Ras4B^G13D^ **•**SOS^cat^ complex (Figure 1F). Furthermore, the differences of fluctuations among individual residues in local regions were revealed by the root-mean-square fluctuations (RMSFs) of Cα atoms of K-Ras4B^G13D^ (Figure 1G) and SOS^cat^ (Figure 1H). Notably, RMSF analysis showed that the switch II region of K-Ras4B^G13D^ exhibited lower flexibilities in response to allosteric K-Ras4BG^13D^−GTP binding, while little distinctions were observed in the switch I and P-loop of K-Ras4B^G13D^ and the SOS^cat^. Taken together, these data indicated higher stability and more constrained conformation of the switch II region in the ternary complex than in the binary complex. This may partially be due to the increased interaction between the catalytic K-Ras4B^G13D^ and SOS^cat^ upon the allosteric K-Ras4B^G13D^–GTP binding.

### 3.2 Principal Component Analysis

**FIGURE 2:**
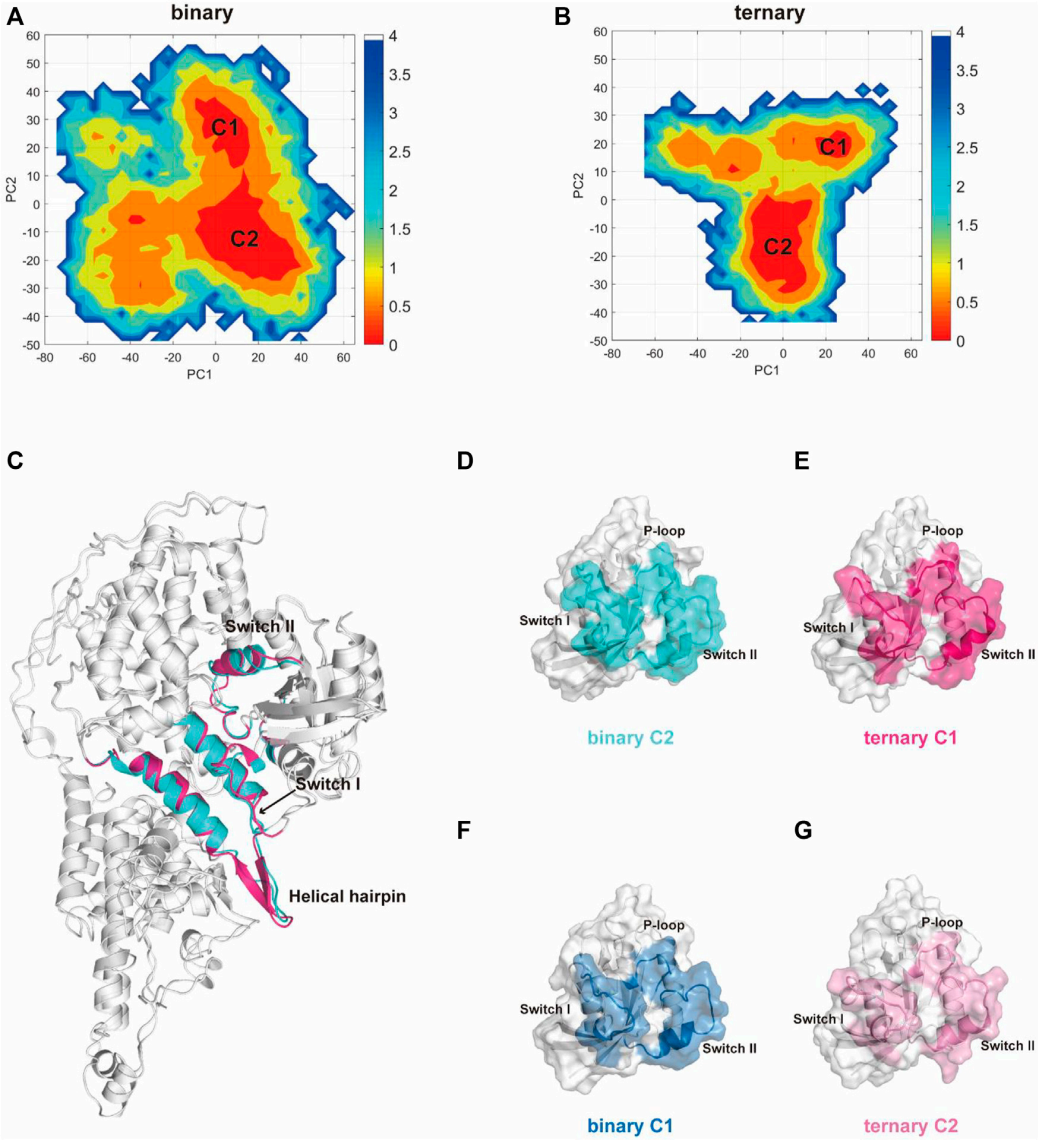
PCA analysis of the K-Ras4B^G13D^•SOS^cat^ complex and the representative structures of clusters C1 and C2 in the two systems. Projection of trajectories along with the first two collective principal components (PC1 and PC2) of K-Ras4B^G13D^•SOS^cat^ in the binary **(A)** and ternary **(B)** systems. **(C)** Cartoon representations of K-Ras4B^G13D^•SOS^cat^ in the binary C2 (cyan) and ternary C1 (hot pink). **(D–G)** Representative structures of K-Ras4B^G13D^ in the binary C2 (cyan), ternary C1 (hot pink), binary C1 (blue), and ternary C2 (light pink).

Subsequently, we used principal component analysis (PCA) for characterizing and comparing the dominant conformations among different systems. It provides more objective information to describe the overall conformational changes in the system. The conformational landscapes of K-Ras4B^G13D^ **•**SOS^cat^ differed obviously in the presence or absence of an allosteric K-Ras4B^G13D^–GTP (Figure 2). Consistent with the trends shown by the RMSD and RMSF results, the conformational landscapes of the ternary system appeared to be more convergent. This indicated that the K-Ras4B^G13D^ **•**SOS^cat^ complex exhibited reduced conformational dynamics upon K-Ras4B^G13D^–GTP binding, further suggesting the regulatory role of K-Ras4B^G13D^–GTP to stabilize the conformational state of K-Ras4B^G13D^ **•**SOS^cat^.

There were two clusters in both binary and ternary systems. Representative structures of each cluster in the two systems were extracted using the cluster analysis, and RMSD of C_α_ atoms was calculated to compare the overall structural dynamics of every cluster. As shown in Table 1, the difference between the RMSD values of K-Ras4B^G13D^ **•**SOS^cat^ in the binary C2 and ternary C1 was the smallest, and their structures shared similar overall conformations through superimposing and comparing the dominant conformers among all clusters (Figure 2C). In addition, the RMSD in the C2 of the ternary system was notably higher than that of the other three clusters, which reflected that a new conformation with considerable changes was formed in the ternary system upon K-Ras4B^G13D^–GTP binding. Significantly, we compared the overall structural similarity of K-Ras4B^G13D^ and found a conspicuous transformation of C2 in the ternary system on the switch I and II regions (Figures 2D–G). These results indicated that K-Ras4B^G13D^–GTP binding initiated the conformational transitions of K-Ras4B^G13D^ **•**SOS^cat^ from the binary system toward the ternary state with significant switch I and II conformational changes. Since both switch I and II regions participate in the interaction of K-Ras4B^G13D^ with SOS^cat^, we further monitored the difference of the K-Ras4B^G13D^ **•**SOS^cat^ interfacial interaction in both binary and ternary systems. Proteins, Interfaces, Structures, and Assemblies (PISA) analyses (Krissinel and Henrick, 2007) of representative structures among every cluster showed that the binding of K-Ras4B^G13D^–GTP significantly strengthened the interaction of SOS^cat^ with K-Ras4B^G13D^ through the formation of more intermolecular hydrogen bonds and salt bridges at the interface in the ternary system (Table 1).

**TABLE 1:** Summary of RMSDs of Cα atoms, salt bridge, and hydrogen bond numbers along the K-Ras4B^G13D^•SOS^cat^ interface in the binary and ternary systems^a^.

	Binary system		Ternary system	
	C1	C2	C1	C2
RMSD	2.6 (0.18)	2.93 (0.20)	2.99 (0.24)	3.27 (0.29)
Salt bridges and hydrogen bonds	25	26	30	31

a Numbers in the parentheses represent SD.

### 3.3 Binding Free Energy Analysis

To evaluate the influence of allosteric K-Ras4B^G13D^–GTP on the binding free energies between the catalytic K-Ras4B^G13D^ and SOS^cat^, the molecular mechanics Poisson–Boltzmann surface area (MM/PBSA) was employed and the binding free energy ( $\Delta G_{binding}$ ) between the catalytic K-Ras4B^G13D^ and SOS^cat^ in both binary and ternary systems were computed. The $\Delta G_{binding}$ values in binary and ternary systems were -146.54 ± 10.28 and -156.22 ± 8.26 kcal/mol, respectively (Table 2). Apparently, the binding free energy between K-Ras4B^G13D^ and SOS^cat^ upon the allosteric K-Ras4B^G13D^–GTP binding increased by 9.68 kcal/mol, which indicated that their interactions in the ternary complex were much stronger than those in the binary system, and the K-Ras4B^G13D^ **•**SOS^cat^ interface in the ternary complex was energetically favored. This may partially result in the enhanced rates of Ras nucleotide exchange activity through increasing the binding affinity of Ras with SOS at the catalytic site.

**TABLE 2:** Binding free energy (kcal/mol) analysis between K-Ras4B^G13D^ and SOS^cat^.^a^

	Binary system	Ternary system
$\Delta E_{vdw}$ ^b^	-138.73 (10.29)	–159.66 (8.72)
$\Delta E_{ele}$ ^c^	-154.08 (22.96)	–106.65 (19.62)
$\Delta E_{PB}$ ^d^	-163.28 (21.01)	128.48 (17.30)
$\Delta E_{nonpolar}$ ^e^	-17.00 (0.87)	–18.39 (0.85)
$\Delta E_{MM}$ ^f^	-292.82 (25.17)	–266.31 (21.90)
$\Delta G_{solv}$ ^g^	-146.29 (20.68)	110.09 (16.82)
$\Delta G_{binding}$	-146.53 (10.28)	–156.22 (8.26)

a Numbers in parentheses represent standard deviations.

b van der Waals force energy contribution.

c Electrostatic force energy contribution.

d Electrostatic component determined by the Poisson–Boltzmann (PB) equation.

e Solvation free energy.

f Total molecular mechanical energy.

g Total solvation energy change.

**FIGURE 3:**
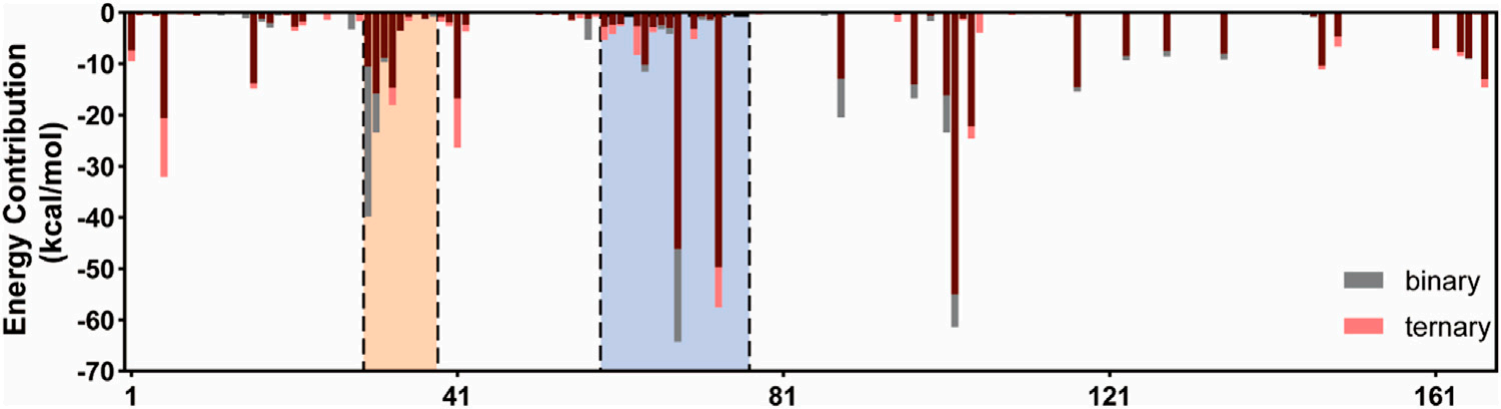
Binding free energy decomposition of the residues of Ras in the K-Ras4B^G13D^•SOS^cat^ complex. The switch I and switch II regions of K-Ras4B^G13D^ are marked with orange and blue backgrounds, respectively.

Furthermore, the $\Delta G_{binding}$ was decomposed into every residue of K-Ras4B^G13D^ to assess per-residue energy contributions for SOS^cat^ binding (Figure 3). The interfacial residues in the K-Ras4B^G13D^ switch I and switch II and α3 helix regions mainly contributed to the binding process. This suggested that the enhanced K-Ras4B^G13D^ **•**SOS^cat^ interactions in the presence of allosteric K-Ras4B^G13D^–GTP could contribute to the stabilization of nucleotide-free form of Ras by SOS.

### 3.4 Dynamics of the Interface Domain Between K-Ras4B^G13D^ and SOS^cat^

**FIGURE 4:**
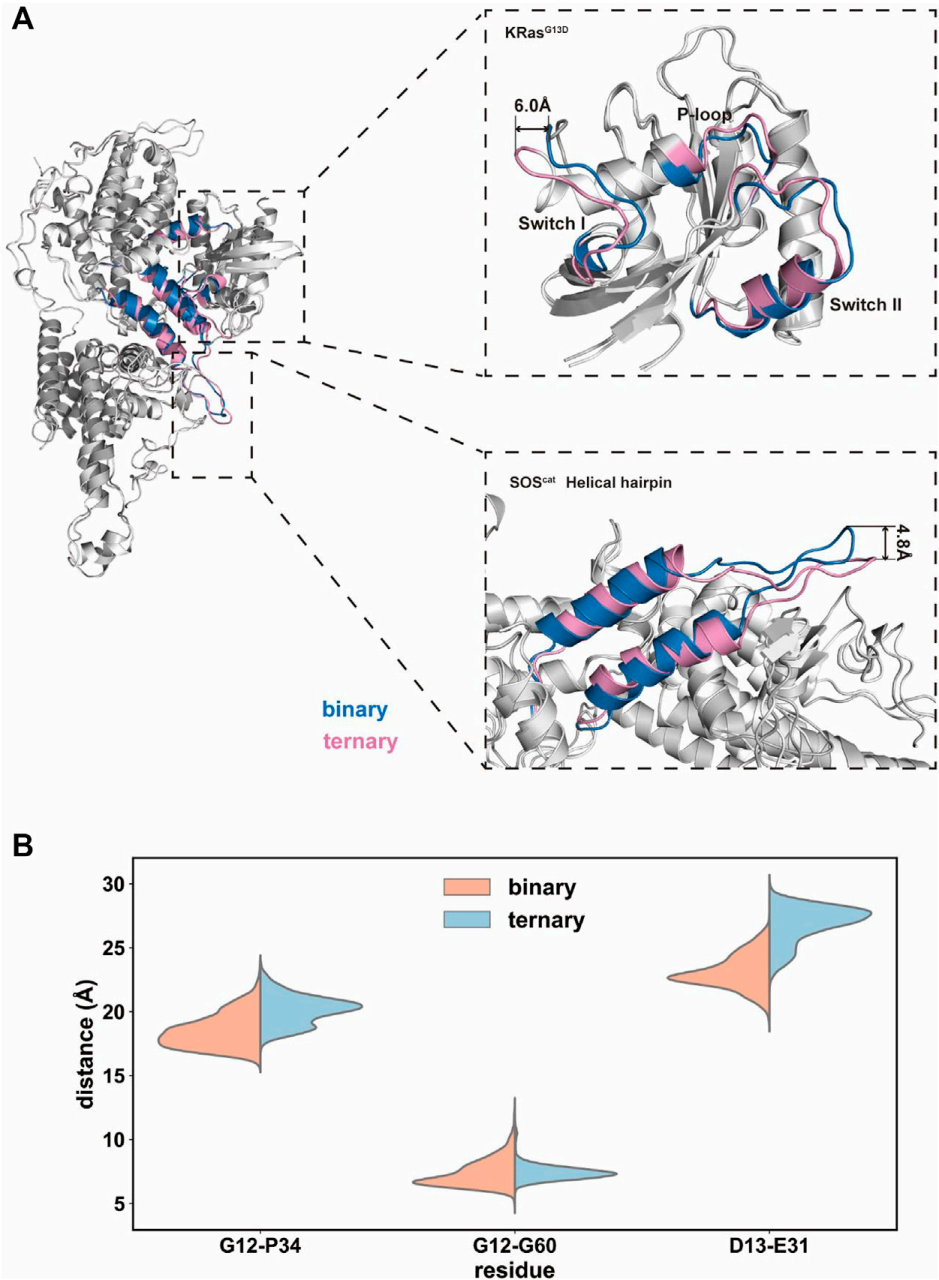
Conformational dynamics of the K-Ras4B^G13D^•SOS^cat^ complex in the binary and ternary systems. **(A)** Representative structure of the K-Ras4B^G13D^•SOS^cat^ complex conformation in the binary (blue) and ternary (pink) systems. **(B)** Distance distributions of Cα atoms between the G12–P34, G12–G60, and G13–E31 residue pairs.

We further extracted the representative structures from the equilibrium stage and superimposed them between the two systems. In the switch I region of K-Ras4B^G13D^, there found the predominant conformational variations between the binary and ternary systems (Figure 4A). The switch I region in the ternary system distinctly stretched away from the rest of Ras and formed significant displacement for ∼6.0 Å. This indicated that the nucleotide binding pocket was expanded in the ternary system, providing space for GDP dissociation. This notion was supported by the distance distributions of Cα atoms among three pairs of inter-residue distances (G12–P34, G12–G60, and G13–E31 residues). To further evaluate the detailed contributions of P-loop, switch I and II regions to the opening of the nucleotide binding pocket, the inter-residue distances were calculated and the probability distributions were shown (Figure 4B). The distances of G12–P34 and G12–G60 pairs described the size of the phosphate binding site and D13–E31 pairs described the size of the ribose binding site (Wang et al., 2021a). The average distances of G12–P34 and D13–E31 pairs significantly increased in the ternary system, while the G12–G60 distance pair showed no obvious distinctions between the two systems. The three pairs of residue distances indicated that the space of the phosphate and ribose binding sites enlarged in the ternary system. The expanded nucleotide binding pocket could result in an increased rate of the nucleotide exchange.

Meanwhile, a prominent difference was found in the conformation of the helical hairpin between the two structures of SOS^cat^ (Figure 4A). The helical hairpin was shifted away from the active site of SOS in the ternary system, and the interdomain distance increased about ∼4.8 Å between the two systems. Consistent with previous evidence, the helical hairpin region of the nucleotide-free Ras–SOS structure constricted the site where SOS attracts the switch II region of nucleotide-free Ras (Freedman et al., 2006; Bandaru et al., 2019). In fact, the combination of Ras–GTP and SOS promoted the rotation and opening of the helical hairpin, thereby freeing the catalytic site where Ras binds to.

### 3.5 Insights Into the K-Ras4B^G13D^•SOS^cat^ Interfacial Residues

Structural comparison and free energy analysis revealed that allosteric K-Ras4B^G13D^–GTP binding altered the principal conformations of switch I and switch II and enhanced the K-Ras4B^G13D^ **•**SOS^cat^ interaction. The detailed differences in the binding process ( $\Delta G_{binding}$ ) caused by the allosteric K-Ras4B^G13D^–GTP mainly due to the elevated van der Waals forces. This suggested that the allosteric K-Ras4B^G13D^–GTP binding led to change the intermolecular interaction patterns between the K-Ras4B^G13D^ **•**SOS^cat^ interfacial residues. We next explored the specific interaction patterns of interfacial residues on the switch I and switch II regions underlying this conformational transformation.

**FIGURE 5:**
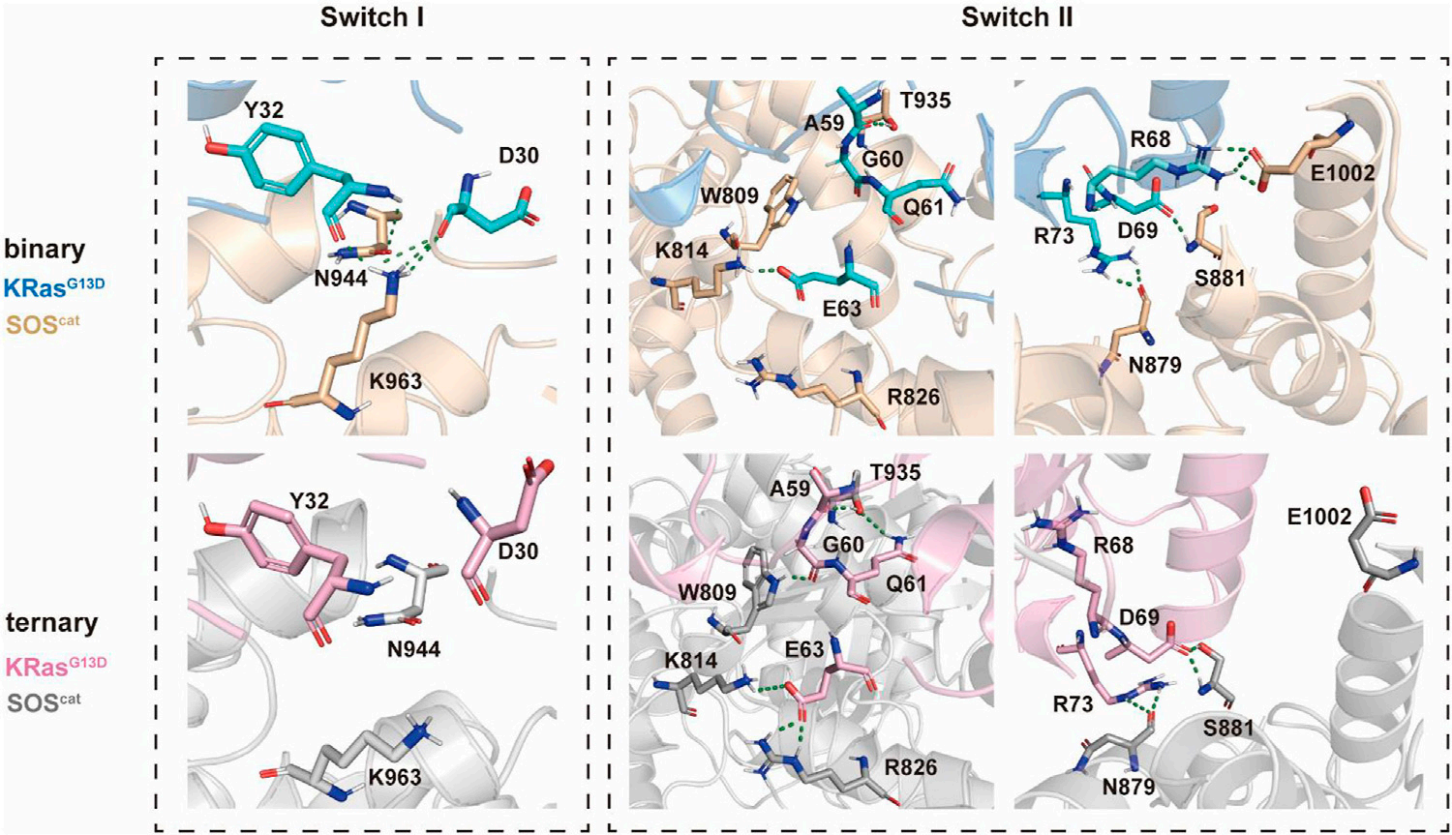
Detailed interaction patterns between K-Ras4B^G13D^ and SOS^cat^ interfacial residues. Hydrogen bonds are marked with green dotted lines.

There were constantly five hydrogen bonds in the binary systems for the interaction between the switch I region and SOS^cat^ domains (Figure 5). Residues Y32 and D30 at the active site on the switch I region of the nucleotide-free K-Ras4b^G13D^ engage in polar interactions with residues N547 and K566 of SOS^cat^ and residues K566 of SOS^cat^, respectively. Nevertheless, upon allosteric K-Ras4B^G13D^–GTP binding, both residues D30 and K566 were shifted away from each other, especially the significant clockwise rotation of K566, which disrupted the hydrogen bond between D30 and K566. This suggested that binding of K-Ras4B^G13D^–GTP impaired the restriction of SOS^cat^ on the switch I region, which may promote the opening of nucleotide binding pocket.

Consistent with the previous findings that switch II provided the main anchor for the interaction of SOS with Ras, we found more hydrogen bonds formed between the switch II region and SOS^cat^ in the ternary system. In the presence of K-Ras4B^G13D^–GTP at the allosteric site of SOS^cat^, residues A59, G60, Q61, and E63 formed significantly more hydrogen bonds with residues T935, W809, K814, and R826 compared to the binary system. Meanwhile, numbers of hydrogen bonds formed at residues R68, D69, and R73 were similar between two systems. The residues of the switch II region underwent significant conformational changes, especially in which residues E63 and R73 rotated counterclockwise and displaced toward the direction of SOS^cat^. These observations indicated that binding of K-Ras4B^G13D^–GTP at the distal site allosterically altered the conformations of key interfacial residues, thereby enhancing the interaction between the switch II region of K-Ras4B^G13D^ and SOS^cat^. The increased affinity of Ras at the catalytic site may promote the nucleotide exchange rate.

### 3.6 Allosteric Signaling Pathways Within K-Ras4B^G13D^•SOS^cat^

#### 3.6.1 Dynamic Cross-Correlation Matrices

**FIGURE 6:**
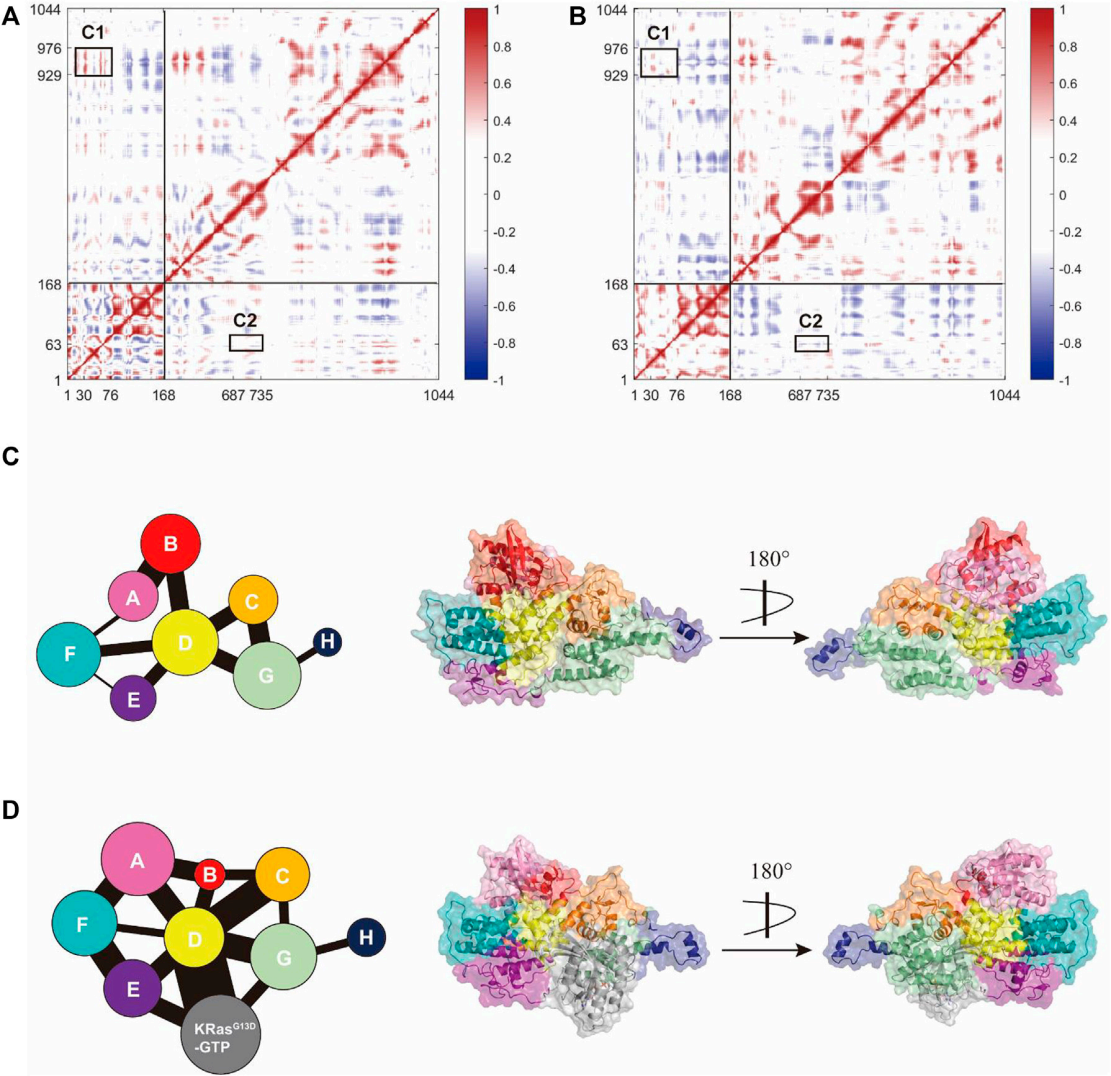
K-Ras4B^G13D^ and SOS^cat^ allosteric interactions. The dynamic cross-correlation matrix of the binary **(A)** and ternary **(B)** systems. Positive regions (red) represent correlated motions, whereas negative regions (blue) represent anticorrelated motions. C1 shows correlations of the helical hairpin domain with the switch I and switch II region, and C2 shows anticorrelated movement of the switch II with the SOS^cat^•K-Ras4B^G13D^–GTP interface residues. Correlated motions with absolute values < 0.3 were neglected and shown in white. Map of the community network in the binary **(C)** and ternary **(D)** systems. Areas of the circles represent the numbers of residues in corresponding communities and the widths of sticks connecting communities represent the intercommunity connections.

We further explored how the signal triggered by K-Ras4B^G13D^–GTP binding at the allosteric site could allosterically regulate the activation of the catalytic K-Ras4B^G13D^ and the interaction patterns between K-Ras4B^G13D^ and SOS^cat^. To determine the dynamic variation of Ras in the two systems, we analyzed the inter-residue correlations using the dynamic cross-correlation matrix calculations. Correlation coefficients were calculated among the related motions between each Cα atom in the whole trajectory composing the dynamic cross-correlation matrices, which reflected the relationship among different domains [(Ni et al., 2021), (Feng et al., 2021)]. As shown in Figures 6A,B, compared to the binary system, the intramolecular anticorrelations of K-Ras4B^G13D^ were weakened in the ternary system, while the correlation of intramolecular motions were slightly strengthened. In each system, C1 represented the correlated movement of the helical hairpin domain of the SOS^cat^ to the switch I and switch II regions of the K-Ras4B^G13D^, while K-Ras4B^G13D^–GTP binding at the allosteric site effectively impaired the C1 correlation. Meanwhile, we investigated the correlation of the switch II region with the interface residues between SOS^cat^ and K-Ras4B^G13D^–GTP. C2 showed the enhanced anticorrelated movement of the switch II with the interface residues. It suggested that the binding of K-Ras4B^G13D^–GTP to the allosteric site of SOS^cat^ resulted in the varied interface residues in the binding site of SOS^cat^ that remotely transmitted to regulate the catalytic activity of K-Ras4B^G13D^.

#### 3.6.2 Community Network Analysis

Subsequently, we focused on the allosteric network from the allosteric K-Ras4B^G13D^–GTP to the catalytic K-Ras4B^G13D^ in both binary and ternary systems. Given that a node was defined within a cutoff distance of 4.5 Å between the two Cα atoms populating at least 75% of the MD trajectory, these residues were categorized into the same community and were regarded as a synergistic functional unit within the protein structure (Foutch et al., 2021). There were eight communities for the K-Ras4B^G13D^ **•**SOS^cat^ complex in both systems and each community was represented by colored circles, whose area was proportional to the amounts of residues contained, connecting by sticks of different width proportional to the value of edge connectivity (Figures 6C,D). In general, their distributions in the two systems were similar, but the eight communities on the K-Ras4B^G13D^ **•**SOS^cat^ interface and within Ras presented prominent differences. In the binary system, the K-Ras4B^G13D^ **•**SOS^cat^ interfacial residues mainly constituted the Communities B and D, while it was formed by the Communities A, B, C, D, and F in the ternary system. This suggested that considerable conformational changes occurred in a portion of residues along the K-Ras4B^G13D^ **•**SOS^cat^ interface upon the allosteric K-Ras4B^G13D^–GTP bidning throughout simulations. Particularly, in the ternary system, the marked change in the K-Ras4B^G13D^ was peeling away of the whole switch I region from the rest of Ras and formed the Community B with a proportion of residues from the SOS^cat^ helical hairpin domain, implying the increased distance between the switch I region and the rest of Ras upon the K-Ras4B^G13D^–GTP binding. It should be noted that the complete switch II region was incorporated into the Community D with partial SOS^cat^ residues, suggesting that they were in close proximity and have more interactions.

On the other hand, the edge connectivity among communities which implied the interactions between communities changed considerably upon K-Ras4B^G13D^–GTP binding. We mainly studied the community cross talk along the K-Ras4B^G13D^ and SOS^cat^ interface. In the ternary system, the binding of K-Ras4B^G13D^–GTP introduced a new strong connection between K-Ras4B^G13D^ and SOS^cat^. The Communities A and B derived from the majority of the residues from K-Ras4B^G13D^ were in direct and strong edge connections with the Communities C and D (main residues from the SOS^cat^ helical hairpin domain). Moreover, the direct information flow between the Communities A and G, representing the partial interfacial residues from SOS^cat^, completely disappeared in the binary system. This indicated that the emerging strong edge connection may strengthen the interaction between K-Ras4B^G13D^ and SOS^cat^, thereby reinforced the allosteric regulation from SOS^cat^ toward the catalytic K-Ras4B^G13D^.

#### 3.6.3 Allosteric Pathway Analysis

Moreover, PISA analyses involving the representative structures of the ternary system showed the critical roles of R694 and W729, I752 and I922 from SOS^cat^ upon the K-Ras4B^G13D^–GTP binding, since they participated in the formation of hydrogen bonds or salt bridges at the interface. We next calculated the optimal and suboptimal pathways, followed by analysis of the potential allosteric relationship from the three interfacial residues in SOS^cat^ down to the switch II region *via* NetworkView plugin in the VMD tool. As listed in Table 3, the pathways from K-Ras4B^G13D^–GTP**•**SOS^cat^ interface toward the switch I and switch II regions in the ternary system presented shorter lengths of the optimal pathway, that is, to say, less residues were involved in the optimal pathway and more suboptimal pathways were formed relative to the binary system. The characterization of these promoted connections highlighted the Community D (αB, αD helix, and αH helix of the Cdc25 domain) and the Community E (especially αC helix of Cdc25 domain) as a core transmission hub. Taken together, these indicated that allosteric K-Ras4B^G13D^–GTP binding exerted an extensive and reinforced allosteric regulation on the catalytic K-Ras4B^G13D^ through the Cdc25 domain of the SOS^cat^.

**TABLE 3:** Allosteric pathway analysis between the SOS^cat^•K-Ras4B^G13D^–GTP interface and the switch I and switch II regions of K-Ras4B^G13D^.

	Length (Å)^a^		Residue^b^		Subopt^c^	
	Binary	Ternary	Binary	Ternary	Binary	Ternary
SOS R694—Ras E63	251	204	9	10	48	50
SOS W729—Ras E63	282	260	11	12	458	2776
SOS I752—Ras D30	434	361	17	12	326	59
SOS I752—Ras I36	454	379	16	11	254	104
SOS I752—Ras D69	436	336	15	10	42	393
SOS I752—Ras R73	456	365	16	9	84	192
SOS I922—Ras E63	214	189	7	6	60	58

a Length of the shortest pathways.

b Numbers of residues involved in the optimal pathways.

c Numbers of the suboptimal pathways.

## 4 Discussion

MD simulations were performed in the binary (K-Ras4B^G13D^•SOS^cat^) and ternary (K-Ras4B^G13D^•SOS^cat^•K-Ras4B^G13D^–GTP) systems to explore the underlying mechanisms driving allosteric activation of the catalytic K-Ras4B^G13D^ through distal binding of K-Ras4B^G13D^–GTP at the allosteric site of SOS^cat^. On the whole, less fluctuations of overall residues and more concentrated conformational landscapes distribution were found in the ternary system. From the perspective of structure, we found that the switch I region of K-Ras4B^G13D^ distinctly stretched away from the rest of K-Ras4B^G13D^ upon K-Ras4B^G13D^–GTP binding, which caused the expanded nucleotide binding pocket. From the perspective of energy, allosteric K-Ras4B^G13D^–GTP binding increased the binding free energy between the catalytic K-Ras4B^G13D^ and SOS^cat^ by enhancing the interactions between the switch II region of K-Ras4B^G13D^ and SOS^cat^. We revealed the detailed mechanism of the activation process of K-Ras4B^G13D^ with structural transformations. Meanwhile, we proposed potential pathways induced by the allosteric K-Ras4B^G13D^–GTP binding to convey the information of K-Ras4B activation over a long-range distance.

SOS-mediated positive feedback had been proposed to dynamically regulate Ras signaling since 2003. The SOS-catalyzed nucleotide exchange by fluorescence spectroscopy showed that H-Ras–GTP markedly increased the rate of nucleotide release from H-Ras stimulated by SOS^cat^ (Vo et al., 2016). Recently, Moghadamchargari et al. found that K-Ras4B^G13D^–GTP can allosterically increase the nucleotide exchange rate of K-Ras4B at the active site >2-fold compared to the K-Ras^WT^–GTP (Moghadamchargari et al., 2021). Moreover, the positive feedback loop exists between H-Ras–GTP and SOS, increases the amplitude and duration of Ras activation after the stimulation of EGF, and leads to the higher activity of downstream proteins (Boykevisch et al., 2006). It was characterized by the sustained EGF-induced ERK phosphorylation and enhanced serum response element (SRE)-dependent transcription (Lu et al., 2016a). These data supported the positive feedback activation of SOS, but previous studies mainly focused on the activation process of SOS by Ras–GTP. Analysis of the resulting structure revealed that the binding of H-Ras^Y64A^–GppNHp at the distal binding site of SOS^cat^ had a significant impact on the conformational change of the Rem domain. This domain rotated by less than 10° relative to the Cdc25 domain, and the rotation changed the affinity of the helical hairpin of SOS^cat^ with the switch I region of nucleotide-free H-Ras^WT^ in the active site (Hall et al., 2001; Boykevisch et al., 2006). This structural feature was verified by mutant residues in the helical hairpin, which was able to stabilize the catalytically competent open conformation (Bandaru et al., 2019). We also observed a similar transformation of SOS^cat^ that the helical hairpin was moved away from the active site of SOS^cat^ upon K-Ras4B^G13D^–GTP binding. This suggested that the nucleotide-free Ras binding to the active site of SOS^cat^ required accommodated space providing by the pulling away of the helical hairpin.

On the other hand, more attention was focused on the process of Ras activation. Liao TJ et al. found the interaction of k-ras4b4B-GTP with SOS1 at the allosteric site induces a local conformation change at the catalytic site, facilitating the accommodation of the inactive Ras (Liao et al., 2018). According to the results, binding of allosteric K-Ras4B^G13D^–GTP may affect the rate-limiting step of the SOS-catalyzed nucleotide change. The switch I region of the catalytic K-Ras4B^G13D^ in the ternary system was distinctly away from the rest of K-Ras4B^G13D^, and the three increased pairwise distances describing the nucleotide binding pocket both suggested the enlarged space of GDP phosphate and the ribose binding site in the ternary system. The opening of nucleotide binding site would weaken the binding affinity of GDP to the K-Ras4B^G13D^, promoting the release of GDP and the subsequent rebinding of GTP to the K-Ras4B active site.

In order to effectively inhibit the activation of Ras catalyzed by SOS, small molecules or peptides that bind to the Ras–SOS interface can be designed based on the Ras–SOS protein–protein interaction (Lu et al., 2016c; Lu et al., 2016d; Ni et al., 2019a; Yang et al., 2021). This may be a potential therapeutic strategy for the treatment of Ras-driven cancer (Ostrem and Shokat, 2016; Ni et al., 2019b; Lu et al., 2021b; Wang et al., 2021b). For instance, it has been reported that nSH3/cSH3 binding peptides, which effectively interrupt the Grb2–SOS interaction, can serve as tumor suppressors (Liao et al., 2020a), (Liao et al., 2020b). However, another interesting phenomenon is that small-molecule Ras•SOS disruptors fail to dissociate K-Ras4B^G13D^•SOS^cat^ complexes. We may explain this evidence from the perspective of binding free energy. Due to the formation of the K-Ras4B^G13D^•SOS^cat^•K-Ras4B^G13D^–GTP ternary complex, allosteric K-Ras4B^G13D^–GTP binding exerted increased binding free energy between K-Ras4B^G13D^ and SOS^cat^. We also provided structural details to explain the observed higher binding affinity of K-Ras4B^G13D^ for SOS^cat^ in response to allosteric K-Ras4B^G13D^–GTP binding. Upon K-Ras4B^G13D^–GTP binding, there were more salt bridges formed between the switch II region and SOS^cat^. Furthermore, the enhanced interaction between K-Ras4B^G13D^ and SOS^cat^ provided a basis for allosteric regulation within the ternary system. The allosteric propagation pathway was found from the K-Ras4B^G13D^–GTP binding site to the K-Ras4B^G13D^ functional region. This indicated that K-Ras^G13D^–GTP at the distal site of SOS^cat^ may regulate K-Ras4B^G13D^ catalytic activity using allosteric modulation.

## Data Availability

The original contributions presented in the study are included in the article/Supplementary Material, further inquiries can be directed to the corresponding authors.
